# Protein Disulfide Isomerase Regulates Endoplasmic Reticulum Stress and the Apoptotic Process during Prion Infection and PrP Mutant-Induced Cytotoxicity

**DOI:** 10.1371/journal.pone.0038221

**Published:** 2012-06-07

**Authors:** Shao-Bin Wang, Qi Shi, Yin Xu, Wu-Ling Xie, Jin Zhang, Chan Tian, Yan Guo, Ke Wang, Bao-Yun Zhang, Cao Chen, Chen Gao, Xiao-Ping Dong

**Affiliations:** 1 State Key Laboratory for Infectious Disease Prevention and Control, National Institute for Viral Disease Control and Prevention, Chinese Center for Disease Control and Prevention, Beijing, People’s Republic of China; 2 Chinese Academy of Sciences Key Laboratory of Pathogenic Microbiology and Immunology, Institute of Microbiology, Chinese Academy of Sciences, Beijing, China; University of Maryland, United States of America

## Abstract

**Background:**

Protein disulfide isomerase (PDI), is sorted to be enzymatic chaperone for reconstructing misfolded protein in endoplasmic reticulum lumen. Recently, PDI has been identified as a link between misfolded protein and neuron apoptosis. However, the potential for PDI to be involved in the pathogenesis of prion disease remains unknown. In this study, we propose that PDI may function as a pleiotropic regulator in the cytotoxicity induced by mutated prion proteins and in the pathogenesis of prion diseases.

**Methodology/Principal Findings:**

To elucidate potential alterations of PDI in prion diseases, the levels of PDI and relevant apoptotic executors in 263K infected hamsters brain tissues were evaluated with the use of Western blots. Abnormal upregulation of PDI, Grp78 and Grp58 was detected. Dynamic assays of PDI alteration identified that the upregulation of PDI started at the early stage and persistently increased till later stage. Obvious increases of PDI and Grp78 levels were also observed in cultured cells transiently expressing PrP mutants, PrP-KDEL or PrP-PG15, accompanied by significant cytotoxicities. Excessive expression of PDI partially eased ER stress and cell apoptosis caused by accumulation of PrP-KDEL, but had less effect on cytotoxicity induced by PrP-PG15. Knockdown of endogenous PDI significantly amended cytotoxicity of PrP-PG15, but had little influence on that of PrP-KDEL. A series of membrane potential assays found that apoptosis induced by misfolded PrP proteins could be regulated by PDI via mitochondrial dysfunction. Moreover, biotin-switch assays demonstrated active *S*-nitrosylted modifications of PDI (SNO-PDI) both in the brains of scrapie-infected rodents and in the cells with misfolded PrP proteins.

**Conclusion/Significance:**

Current data in this study highlight that PDI and its relevant executors may function as a pleiotropic regulator in the processes of different misfolded PrP proteins and at different stages during prion infection. SNO-PDI may feed as an accomplice for PDI apoptosis.

## Introduction

Prion disorders, or transmissible spongiform encephalopathies (TSEs), are fatal neurodegenerative diseases affecting humans and many species of animals, for example Creutzfeldt-Jakob disease (CJD) in humans, scrapie in sheep and goat, and bovine spongiform encephalopathy (BSE) in cattle. Prion-related disorders share common neuropathology characteristics, such as spongiform degeneration, extensive neuronal loss and the accumulation of PrP^Sc^, a misfolded and protease-resistant form of the normal prion protein (PrP^C^) [1]. Although the mechanism of PrP^Sc^ pathogenesis is still controversial, mounting evidences suggests that perturbations in endoplasmic reticulum (ER) homeostasis or mitochondrial dysfunction induced by PrP^Sc^ or misfolded prion proteins may contribute to cell death or neurodegenerative pathology in prion disease [2], [3], [4].

When ER homeostasis is perturbed, ER stress triggers the survival pathway, unfolding protein response (UPR), which is associated with upregulation of ER-derived chaperones and protein-folding enzymes leading the misfolded proteins undergoing the process of degeneration [5], [6]. Conventionally, activation of UPR increase pro-survival effects including an increased expression of the glucose-related protein (Grp) family, such as Grp78/Bip, and chaperones with protein disulfide isomerase (PDI) -like activities, such as PDI and Grp58 (also known as PDIA3) [7]. In the terminal stage of the scrapie-infected murine model, upregulation of Grp58 is mainly associated with an ER stress response. Moreover, Grp58 and its structural homolog PDI have neuroprotective activities against ischemia [8]. On the other hand, when ER homeostasis cannot be restored, the pro-apoptotic process is irreversiblely induced, executed by ER-resident caspase-12 or caspase-3 in the cytoplasm [9]. Interestingly, it was revealed that PDI and Grp58 were also involved in apoptosis induced by misfolded proteins [10]. Although the PDI-mediated apoptotic pathway seems to be specific for misfolded proteins, the role of PDI in prion-related diseases or misfolded PrPs remains unclear.

PDI is a target of nitrosative stress, which results in *S*-nitrosylation of normal PDI. *S*-nitrosylated PDI (SNO-PDI) has been found in the brains of patients with Parkinson’s disease and Alzheimer’s disease, which are commonly characterized by intracellular or extracellular accumulation of misfolded proteins [11]. Under conditions of severe nitrosative stress, SNO-PDI can prolong the UPR, thus contributing to aberrant protein accumulation and cell damage [11]. In the model of Huntington’s disease, PDI has been identified as a regulator for a cell death pathway [10]. Chemicals that limit the activity of PDI, or shRNAs that down-regulate PDI expression, ultimately provide a protective benefit for cells displaying misfolded protein. However, the potential alteration of SNO-PDI in prion diseases remains to be documented.

In the present study, we investigated the functional relationship between PDI and misfolded PrP proteins in the induction of ER stress, mitochondria dysfunction and the apoptotic process, by measuring the potential changes of relative markers either in scrapie-infected brain tissues or in cell models transiently expressing two human PrP mutants, PrP-KDEL, which can be retained in ER with an artificial KDEL tag mimicking PrP mutants within the transmembrane region [12], and PrP-PG15, which contains 10 extra-octarepeat insertions mimicking human genetic prion-disease- associated PrP insertions within the octarepeat region. Our results demonstrated abnormally increased PDI family members in the brains of scrapie-infected hamsters and in cells expressing misfolded PrP mutants. Overexpression of misfolded PrP in cells upregulated the level of PDI seems to be a two-character event. Excessive expression of PDI eased ER stress and cell apoptosis caused by accumulation of the PrP mutant in ER, but had less effect on cytotoxicity induced by PrP mutants with extra-octarepeat insertions. Knockdown of endogenous PDI significantly amended cytotoxicity caused by PrP with extra-octarepeat insertion, but had little influence on the cytotoxicity induced by PrP mutants anchored in the ER. We also demonstrated that mitochondrial dysfunction contributed to the PrP-mutants-induced apoptotic effect, and *S*-nitrosylation of PDI plays an essential role in PDI-mediated cytotoxicity.

## Materials and Methods

### Antibodies

The following antibodies were used in this study, including anti-actin mouse monoclonal antibody (mAb), anti-cytochrome *c* rabbit polyclonal antibody (pAb), anti-VDAC1 mouse mAb, anti-Bcl-2 pAb, anti-Bax mAb and anti-PDI pAb (Santa Cruz (USA); anti-pro-caspase-3, anti-pro-caspase-12, anti-GRP58, and anti-GRP78/Bip (Abcam, Hong Kong); PrP specific mAb 3F4 (Dako, Denmark); HRP-conjugated anti-rabbit or anti-mouse immunoglobulin G (Boehringer, Germany); and FITC-conjugated anti-mouse IgG and Alexa Fluor 568 goat anti-rabbit IgG (Invitrogen, USA).

### Ethics Statement

The use of animal specimens in this study was approved by the Ethical Committee of the National Institute for Viral Disease Prevention and Control, China CDC under protocol 2009ZX10004-101. All signed informed consents forms were collected and stored by the China CJD Surveillance Centre. All Chinese golden hamsters were maintained under clean grade. Housing and experimental protocols were in accordance with the Chinese Regulations for the Administration of Affairs Concerning Experimental Animals.

### Preparation of Brain Tissue Samples

Four Chinese golden hamsters (innoculated intracerebrally with hamster-adapted scrapie agent 263K ) and three normal hamsters were enrolled in this study. Brain homogenates (10%, w/v) were prepared based on the protocol described previously [13]. Briefly, brain tissues were homogenized in lysis buffer (100 mM NaCl, 10 mM EDTA, 0.5% Nonidet P-40, 0.5% sodium deoxycholate, 10 mM Tris, pH 7.5) containing a mixture of protease inhibitors. The tissue debris was removed with low-speed centrifugation at 2,000 *g* for 10 min and the supernatants were collected for further study.

### Plasmids and Cell Transfection

The full-length human PDI complementary DNA (cDNA ) was amplified by a polymerase chain reaction (PCR ) protocol, with the specific primers (5′-CGCGGATCCATGCTGCGCCGCGCTCTGCT-3′, with a B*am*HI site underlined) and (5′-CCGGAATTCTTTACAGTTCATCTTTCACAGCTT-3′, with an E*co*RI site underlined) from the reverse transcripts of the RNA extracted from cultured 293T cells. PCR was conducted under the following conditions, 94°C for 30s, 55°C for 30s and 72°C for 30s, for a total of 30 cycles. The amplicon was digested with B*am*HI and E*co*RI and cloned into vector pcDNA3.1, generating plasmid pcDNA-PDI. Recombinant plasmids pcDNA-PrP-WT (previously named as pcDNA-PrP-PG5), pcDNA-PrP-G114V, pcDNA-PrP-PG14, pcDNA-PrP-PG-15, pcDNA-PrP-KDEL and pcDNA-PS1-KDEL were constructed previously [12], [14].

Small interfering RNA oligonucleotides (oligo) targeting human PDI RNA were synthesized by GenePharma (Shanghai, China) as followes: PDI-1003-sense strand: 5′-GGCAAACUGAGCAACUUCATT-3′ and PDI-1003-antisense strand: 5′-UGAAGUUGCUCAGUUUGCCTT-3′. The transfection procedure was performed as previously described [12]. Briefly, monolayer cell line 293-T was maintained in DMEM (Gibco BRL, USA). Cells were plated onto 12-well or 96-well plates (Corning, USA) 24 h before transfection. Various recombinant plasmids or RNA oligo (1 µg DNA or 40 pmol RNA oligo per well in 12-well plates; 0.1 µg DNA or 5 pmol RNA oligo per well in 96-well plates ) were transfected with FuGENE^®^ HD reagent (Roche, Switzerland) according to the manufacturer’s instructions. A GFP expressing plasmid, pcDNA-GFP, was used to estimate the efficacy of transfection. 48 h post-transfection, cells were harvested in RIPA lysis buffer (Beyotime Institute of Biochemistry, China) by cell scraper.

### Isolation of Cytosolic Fraction

Cell fractions were isolated by a commercial Qproteome mitochondria isolation kit (Qiagen, Germany). Briefly, all grouped cells were stripped by trypsin digestion, and washed with 0.9% sodium chloride solution once. Aliquots of the lysis buffer containing protease inhibitor solution were added in order to disrupt the plasma membrane mildly. The lysate was mixed with ice-acetone and maintained at −20°C for 1 h. After Centrifuging at 12000 g for 10 min, the pellet was resuspended carefully with ice-cold disrupting buffer using a syringe with blunt-ended needle carefully. The preparation was spun at 6000 g for 10 min and the pellet was collected as the fraction of mitochondria.

### SDS-PAGE and Western Blot Analysis

Aliquots of each sample were prepared for 15% sodium dodecyl sulfate-polyacrylamide gel electrophoresis (SDS-PAGE) by addition of 5 times loading buffer. Samples with loading buffer were heated for 10 min, following electrophoresis; the fractionated proteins were transferred to nitrocellulose (NC) membranes (Whatman, USA) by the semi-dry method in transfer buffer. After there were blocked with 5% non-fat milk powder in TBST buffer, membranes were incubated with the individual primary antibodies at room temperature for 2 h. Membranes were subsequently incubated with different HRP-conjugated secondary antibodies and reactive signals were visualized using an enhanced chemiluminescence kit (Amersham-Pharmacia Biotech, USA).

### Mitochondrial Membrane Potential Assay

The situations of the mitochondrial transmembrane transition in live cells were determined with the use of a commercial kit (MitoCapture™ Mitochondrial Apoptosis Detection kit, BioVision), following the manufacturer’s instructions. Growing cells (about 60% confluence) were transiently transfected with various recombinant plasmids or RNA oligo. Cells were collected 30 h after transfection and resuspended in 1 ml diluting MitoCapture solution, followed by incubation at 37°C for 20 min. After a short spinning, the cells were resuspended in 1 ml pre-warmed incubation buffer. Cells were mounted onto glass slips and examined under a fluorescence microscope (Olympus BX51, Japan).

The ratios of positive cells with green fluorescence were determined with flow cytometry that monitored the signal of FITC and PI channels simultaneously. Briefly, 48 h after transfection, cells were harvested and resuspended in 1 ml diluting MitoCapture solution, followed by incubation at 37°C for 20 min. Samples were analyzed on a FACScan flow cytometer (Becton Dickinson, Oxford, UK). Fluorescence was measured through a 530/30 band filter (FL-1) to monitor FITC and through a 585/42 band filter (FL-2) to monitor PI uptake.

### Caspase-3 Activity Assay

Caspase-3 activity was measured according to a colorimetric protocol based on the manufacturer’s instructions (Calbiochem, USA). Briefly, 1×10^6^ cells were transfected with various constructs or challenged with PDI-specific siRNA, 48 h later, cells were harvested and cell extracts were prepared. Under an ice bath, Ac-DEVE-pNA used as the substrate for activated caspase-3 was mixed with aliquots of cells extracts. Reactions were conducted in 96-well plates at 37°C for 120 min in the dark. Absorbance was determined with a microELISA plate reader (Thermo MK3, USA) at 405 nm. A recombinant human caspase-3 provided in the kit was used as positive control.

### Cell Viability Assay

Cell viability was determined with the use of a CCK-8 cell counting kit (Dnjindo, Japan). Briefly, cells were treated according to the assigned protocol and mixed with 10 µl CCK-8 reagent in each well at 37°C for 1 h. Absorbance was measured at 450 nm with a spectrophotometer. Each experiment was performed in triplicate and repeated at least three times.

### Immunocytochemistry

Cells adhered to coverslips were fixed with 4% paraformaldehyde (PFA) at room temperature for 10 min and permeabilized with 0.5% Triton X-100 for 5 min. Nonspecific binding was minimized by incubation in the blocking solution (10% bovine fetal serum in PBS) at 37°C for 1 h. Cells were incubated with the 1∶500 diluted PrP mAb 3F4 and PDI pAb at 37°C for 2 h. the cells were washed with PBS, then incubated with anti-mouse and anti-rabbit antibodies conjugated with FITC 488/Alexa Fluor 568 at 37°C for 1 h. Cell nuclei were fluorescently stained with DAPI dye. For Golgi staining, cells incubated with the 1∶500 diluted PrP mAb 3F4 and anti-mouse antibody conjugated with FITC-488 were further stained with DAPI dye and Golgi-Tracker Red (Beyotime Institute of Biochemistry, China) at 4°C for 30 min. The images of cells were visualized with the use of a confocal microscopy (Leica ST2, Germany).

### Biotin-switch Assay for Detecting *S*-nitrosylated Protein

*S*-nitrosylation of proteins in brain tissues and in cell lysates was performed with a commercial detection assay kit (Cayman Chemical, USA) according to the manufacture’s instructions. Briefly, cell lysates and brain tissue homogenates were mixed with the blocking buffer, gently agitating the mixture at 4°C for 30 minutes to block free thiol groups. The blocking agent was replaced with ice-cold acetone precipitation at −20°C, and the nitrosothiols were reduced to thiols with a reducing reagent. The newly formed thiols were labeled with a biotinylating reagent. The total biotinylated proteins were immunoprecipitated with streptavidin-agarose beads. and SNO-PDI was detected with Western blots.

### Statistical Analysis

Based on quality control, any experimental data used for quantitative analysis were repeated at least three times. Data were analyzed by a parametric *T* test (two tailed), and *P* values less than 0.05 were considered to be statistically significant. When more than two groups were analyzed, an ANOVA test with the *SPSS* 17.0 statistical package was also used to estimate statistical significance. All quantitative analyses of immunoblot images were carried out using software Image J.

## Results

### Upregulation of PDI Proteins during Scrapie Experimental Infection

**Figure 1:**
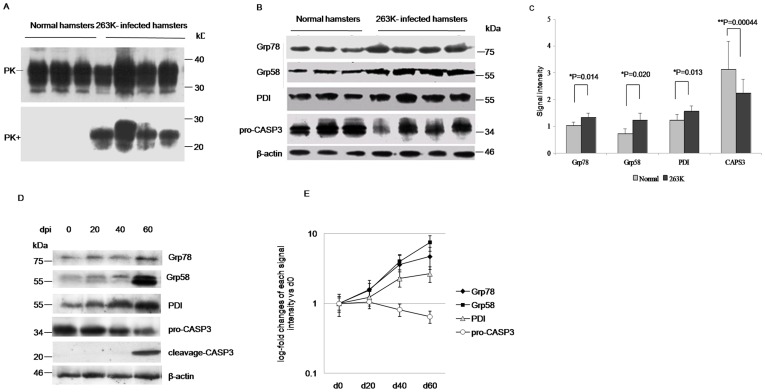
Comparative analysis of the levels of PDIs in the brain tissues of normal and 263K-infected hamsters. A. Western blots. The infected hamsters were collected about 60 to 70 dpi. Same amounts of individual brain homogenate were loaded in 15% SDS-PAGE and immunoblotted with PrP specific mAb 3F4. PK+: with PK (50 µg/ml); PK-: without PK. B. Western blots of Grp78, Grp58, PDI and pro-caspase3. C. Quantitative analysis of each gray numerical value of Grp78, Grp58, PDI and pro-caspase3 vs that of individual β-actin. The average values are calculated from four infected hamsters or three normal hamsters and presented as mean ± SD. Statistical differences compared with controls are illustrated. D, Dynamic analyses of Grp78, Grp58, PDIs, pro-caspase3 and cleveage-caspase3 in the brain tissues of scrapie agent 263K-infected hamsters collected on 0, 20, 40 and 60 dpi during incubation period with Western blots. E. Log-fold changes of the gray values of Grp78, Grp58, PDIs, pro-caspase3 in the 263K-infected hamster’s brains collected on 20, 40 and 60 dpi vs that of 0 dpi.

To assess the possible change of PDI in the brains with prion disease, the levels of some PDI family members in hamsters brain tissues infected with scrapie strain 263K were evaluated with individual Western blots. Obvious PK-resistant PrP signals (PrP^res^) were detected in infected hamsters brains collected at the terminal stage, which were collected about 60 to 70 days post-inoculation (dpi) (Fig. 1A). Two candidates from the PDI family, Grp58 and PDI as well as another ER-anchored chaperone Grp78/Bip were individually detected with the dependable specimens. Significant upregulation in the expression levels of Grp78/Bip, Grp58, and PDI were detected in the samples of infected hamsters (Fig. 1B). Mean relative gray values of three ER makers in scrapie-infected animals showed statistical differences compared with that of normal controls, after they were normalized with individual data from β-actin (Fig 1C). Additionally, the amounts of pro-Caspase3 (pro-CASP3) in the brains of the infected hamsters were lower than that of controls (Fig 1, B and C), revealing signs of apoptosis.

To address the dynamic alteration of PDIs in the brains of scrapie experimental hamsters during the incubation period, the levels of various PDIs in the agent 263K-infected hamster brain samples collected on the 20^th^, 40^th^ and 60^th^ dpi were comparatively analyzed with Western blots. Signals from Grp78, Grp58 and PDI levels were clearly upregulated on 20 dpi, increasing until reaching a maximum on 60 dpi, whereas the levels of pro-CASP3 were decreased from 40 dpi and the signal of cleavage-CASP3 was observed on 60 dpi (Fig 1D and E). These results illustrate that UPR activation and increased expression of PDIs initially begin at the early stage of scrapie infection. Even at the terminal stage of the scrapie-infected animals, PDIs are still keep to be upregulated, accompanied by activation of caspase-3.

### Abnormality of Misfolded PrP Mutants in the Cell Models

**Figure 2:**
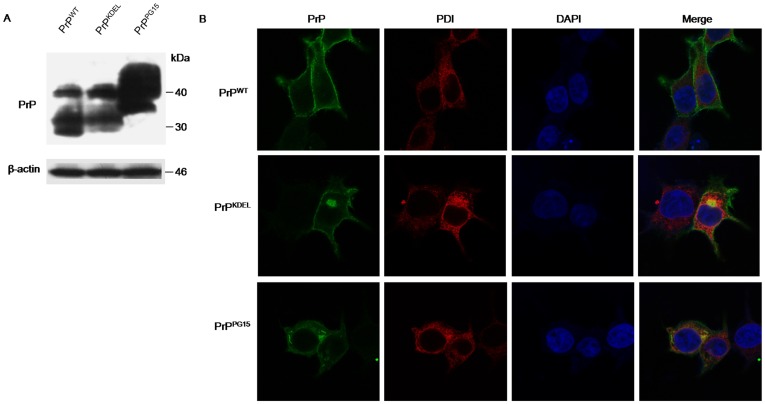
Expressions of transiently transfected PrP constructs and co-localizations of the expressed PrPs with PDI in 293-T cells. A. Western blots of the expressions of wild-type PrP (PrP^WT^) and PrP mutants (PrP^KDEL^ and PrP^PG15^). B. Immunocytochemial assays of the cells transfected with various PrP constructs 48 h after transfection. The images of PrP (green), PDI (red), DAPI (blue) and merge are monitored under a confocal microscopy and indicated above. (×1000).

Our previous studies demonstrated that transient expressions of the PrP construct retained in ER (PrP-KDEL) and PrP with extra-octarepeats insertions [15] on the cultured cells induced ER stress and cell apoptosis. To obtain more detailed insight into the involvement of PDI in the prion-associated abnormality, recombinant plasmids expressing wild-type PrP (pcDNA-PrP-WT), PrP with additional ER-anchored peptide (pcDNA-PrP-KDEL) and PrP with 10 extra-octarepeats (pcDNA-PrP-PG15) were individually introduced into 293-T cells that were confirmed without detectable endogenous PrP^C^ in routine Western blot assays [15]. Clear PrP-specific signals were observed in the cells receiving the various PrP-expressing plasmids, in which wild-type PrP (PrP^WT^) and PrP with additional ER-anchored peptide (PrP^KDEL^) migrated at the same position (ranging from 25 to 35 kDa), while PrP with ten extra-octarepeats (PrP^PG15^) at the position from 35 to 45 kDa (Fig 2A).

To see the subcellular locations of the expressed PrPs as well as their co-locations with cellular endogenous PDI, cells transiently transfected with different PrP constructs were subjected to immunocytochemical assays. Confocal microscopy identified that the signal for PrP^WT^ (green) mainly distributed in the plasma membrane, which did not co-localized with the signal for PDI in cytoplasm (red) (Fig. 2B, top). The expressed PrP^KDEL^ displayed on plasma membrane and in cytoplasm, but mainly concentrated as plaque-like structures in the cytoplasm, which co-localized well with the PDI positive signal on the ER (Fig. 2B, middle ). Signals for PrP^PG15^ were observed both on plasma membrane and in cytoplasm, and among them, the plaque-like structure accumulated on the ER and in the perinuclear compartment overlapped with the signal for PDI (Fig. 2B, bottom).

It has been suggested that disease-causing mutated PrP will experience post-ER metabolism, trafficking to the Golgi apparatus [16]. To see the relationship between expressed PrPs and the Golgi body, cells receiving various PrP constructs were double-stained with PrP-specific mAb and Golgi-Tracker Red. The expressed PrP^WT^ appeared not tp overlap with signals from the Golgi body, whereas both expressed PrP mutants, especially PrP^KDEL^, partially overlapped with Golgi-positive signals (Supplemental Fig S1). It seems that expressed PrP mutants have a stronger disposition to accumulate in cytoplasm organelles, such as reticulum organelles, the perinuclear inclusion compartment and Golgi apparatus, and to co-localize with PDI, which may indicate an abnormal posttranslational modification and maturation process.

### Misfolded Prion Proteins Induced Cell Death through ER Stress and Activation of Caspases

**Figure 3:**
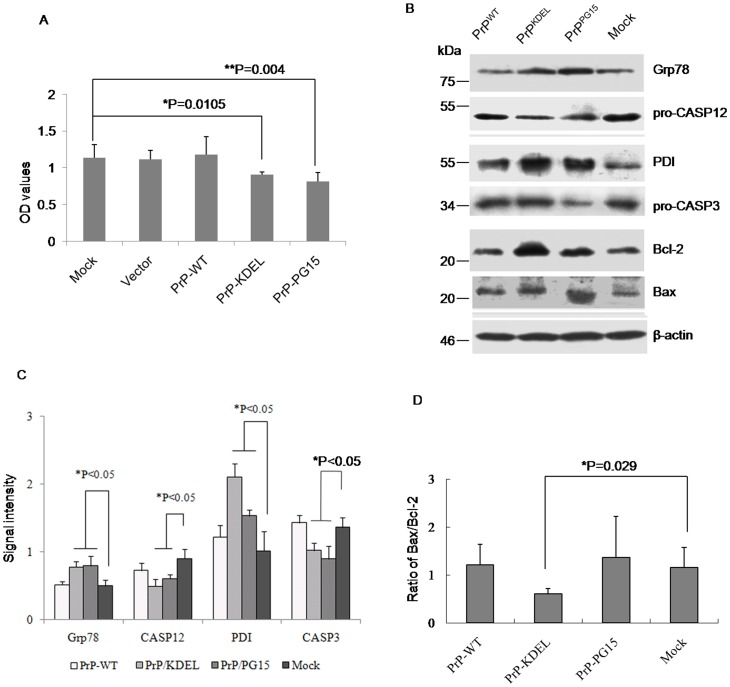
Influences of expression of various PrP constructs on the cell viability and the relevant cellular factors. A. Cell viabilities assayed by CCK-8 kit. Each group is assigned to three repeating parallel units and measured with a spectrophotometer under 450 nm. The results are calculated from three independent tests and indicated as mean ± SD. Statistical differences compared with controls are illustrated as *P<0.05. B. Western blots. Cell lysates were separated in 15% SDS-PAGE and the specific immunoblots for Grp78, pro-caspase12, PDI, pro-caspase3, Bcl-2 and Bax were detected with individual antibodies. C. Quantitative analysis of each gray numerical value of Grp78, pro-caspase12, PDI, pro-caspase3 vs that of individual β-actin. The results are calculated from three independent tests and presented as mean ± SD. Statistical differences compared with controls are illustrated as *P<0.05. D. The relative densities of Bcl-2 and Bax specific bands normalized to β-actin are presented as ratio of Bax/Bcl-2. Statistical differences compared with controls are illustrated.

Previous studies have repeatedly confirmed that transient expressions of PrP-KEDL and PrPs with extra-octarepeat insertions cause cell apoptosis and pathological abnormality in animal models or cell lines [17], [18], [19]. Under these experimental conditions, expressions of PrP-KEDL and PrP-PG15 in 293-T cells significantly decreased cell viability (Fig. 3A). To see the changes in cellular agents related to ER stress and apoptosis, cells receiving different PrP constructs were harvested 48 h posttransfection and levels of individual molecules were evaluated by Western blots. In line with the observation in scrapie-infected hamsters, the levels of two ER-chaperone members, Grp78 and PDI, were markedly increased in cells expressing PrP-KEDL and PrP-PG15, whereas that in cells expressing wild-type PrP remained unchanged compared with the mock (Fig. 3B and C). Consistent with that, an executor of ER apoptosis, caspase-12, was activated by expressions of abnormal PrPs, which possessed a significant decrease in its precursor (Fig. 3C). Meanwhile, levels of pro-caspase-3 were downregulated, whereas that of Bax were upregulated in cells expressing those two PrP mutants, representing activation of the apoptotic executors caspase-3 and Bax (Fig. 3C and D). Those results indicate that ER stress and caspase activation contribute to PrP-KEDL and PrP-PG15-induced cytotoxicity.

To exclude the possible influence of the KDEL tag, a mammalian-expressing plasmid encoding full-length presenilin 1 (PS1) with an extra amino acid KDEL at the C-terminus (PS1-KDEL) was introduced into 293-T cells. As expected, expression of PS1-KDEL did not induce detectable cytotoxicity (Supplemental Fig S2A) and remarkable changes of the tested biomarkers for ER-stress and apoptosis (Supplemental Fig S2B and C). To profoundly present the associations of those two PrP mutants with natural familial CJD (fCJD) related PrPs, two human mutated PrP constructs, PrP-G114V with a glycine-to-valine exchange at the position of amino acid.114 within the transmembrane region and PrP-PG14 with nine extra-octarepeat insertions, were transiently expressed in 293-T cells. In line with the previous results [12], [15], obvious cytotoxicities were observed in the cells expressing PrP-G114V and PrP-PG14 (Supplemental Fig S2A). Western blot assays showed significant increases in levels of PDI and Grp78 and decreases in that of pro-CASP12 and pro-CASP3 in cells expressing PrP-G114V and PrP-PG14, which were similar to that of PrP-KEDL (Supplemental Fig S2B and C).

### Overexpression of PDI Functioned as a Survival Factor for Cells Expressing PrP Mutants Accumulated in the ER

**Figure 4:**
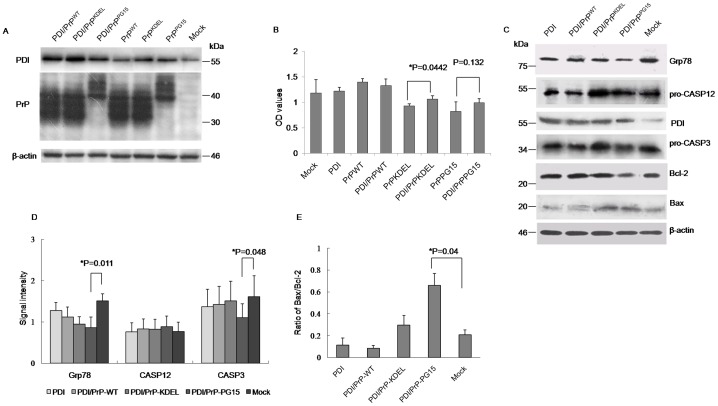
Influences of overexpression of PDI on PrP mutants-induced cytotoxicity and the alterations of relevant cellular factors. A. Evaluation of the influence overexpressing PDI on the expressions of various PrP constructs by Western blots. Cell lysates were separated in 15% SDS-PAGE and the specific immunolots for PDI and PrP were reacted with individual antibodies. B. Cell viabilities assayed by CCK-8 kit. Each group is assigned to three repeating parallel units and measured with a spectrophotometer under 450 nm. The results are calculated from three independent tests and indicated as mean ± SD. Statistical differences compared with controls are illustrated as *P<0.05. C. Western blots. Cell lysates were separated in 15% SDS-PAGE and the specific immunolots for Grp78, pro-caspase12, PDI, pro-caspase3, Bcl-2 and Bax were detected with individual antibodies. D. Quantitative analysis of each gray numerical value of Grp78, pro-caspase12, pro-caspase3 vs that of individual β-actin. The results are calculated from three independent tests and presented as mean ± SD. Statistical differences compared with controls are illustrated. E. The relative densities of Bcl-2 and Bax specific bands normalized to β-actin are presented as ratio of Bax/Bcl-2. Statistical differences compared with controls are illustrated.

To see the potential role of PDI in misfolded PrP-induced cytotoxicity, a PDI-expressing plasmid was introduced into the cell model to create a situation of overexpression of PDI. Western blots revealed that transfection of pcDNA-PDI increased cellular PDI levels but did not influence levels of expressed PrPs when co-transfected with different PrP-expressing plasmids (Fig 4A). CCK-8 assays showed that transient expression of PDI alone did not affect cell growth, which revealed cell viability similar to mock and the preparation of wild-type PrP (Fig 4B). Co-transfection of the plasmid-expressing PDI and PrP mutants reversed to a modest degree PrP mutants-induced cytotoxicity, but showed distinct effectiveness, in which overexpression of PDI rescued PrP^KDEL-^ induced cytotoxicity with statistical significance but seemed to have less effect on PrP^PG15^-induced cytotoxicity (Fig. 4B). Western blots of cell lysates identified that, in the preparation co-expressing PDI and PrP^KDEL^, the levels of Grp78, pro-caspase-12, and pro-caspase-3 recovered to a degree comparable to the control, whereas levels of pro-caspase-3 in the preparation co-expressing PDI and PrP^PG15^ were obviously lower than the controls (Fig 4C and D). Additionally, levels of Bax in those two preparations were still higher than controls (Fig. 4E). It seems that overexpression of PDI in cultured cells sufficiently releases ER stress and antagonizes apoptosis induced by misfolded PrP mainly accumulated in the ER, but has a limited effect on the apoptosis of other organelles.

### Knockdown of Endogenous PDI Rescued Apoptosis Induced by PrP Mutants with Octarepeat Insertions

**Figure 5:**
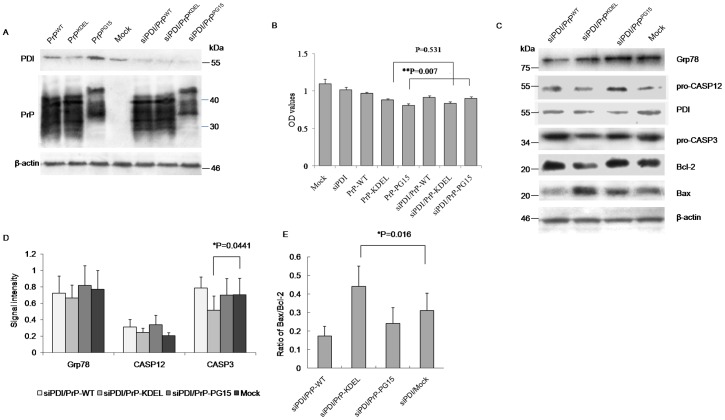
Influences of knockdown of endogenous PDI on PrP mutants-induced cytotoxicity and the alterations of relevant cellular factors. A. Evaluation of the influence down-regulating PDI on the expressions of various PrP constructs by Western blots. Cell lysates were separated in 15% SDS-PAGE and the specific immunolots for PDI and PrP were reacted with individual antibodies. B. Cell viabilities assayed by CCK-8 kit. Each group is assigned to three repeating parallel units and measured with a spectrophotometer under 450 nm. The results are calculated from three independent tests and indicated as mean ± SD. Statistical differences compared with controls are illustrated as **P<0.001. C. Western blots. Cell lysates were separated in 15% SDS-PAGE and the specific immunolots for Grp78, pro-caspase12, PDI, pro-caspase3, Bcl-2 and Bax were detected with individual antibodies. D. Quantitative analysis of each gray numerical value of Grp78, pro-caspase12, pro-caspase3 vs that of individual β-actin. The results are calculated from three independent tests and presented as mean ± SD. Statistical differences compared with controls are illustrated. E. The relative densities of Bcl-2 and Bax specific bands normalized to β-actin are presented as ratio of Bax/Bcl-2. Statistical differences compared with controls are illustrated.

To determine the effectiveness of removal of PDI on the release of ER stress caused by expressions of PrP mutants, PDI-specific siRNAs were synthesized and introduced into 293-T cells. Evaluation of expression levels of cellular PDI by Western blot verified a clearly downregulated situation after transfected PDI-specific siRNA, caused roughly 40 to 60% reduction in expression of endogenous PDI, but did not affect PrP levels when co-transfected with different PrP-expressing plasmids (Fig 5A). CCK-8 assays did not reveal remarkable cytotoxicity on cultured cells after introduction of PDI-specific siRNA (Fig 5B). Co-expression of PrP^KEDL^ with PDI-specific siRNA induced slightly lower cell viability than the expression of PrP^KEDL^ alone, but without statistical difference (Fig 5B). However, co-expression of PrP^PG15^ with PDI-specific siRNA did not aggravate, but improved PrP^PG15^-induced cytotoxicity (Fig 5B). Western blots revealed downregulated pro-caspase 3 and Bcl-2, and upregulated Bax in cells receiving plasmid-expressing PrP^KEDL^ and PDI-specific siRNA, and levels of molecules tested in cells receiving PrP^PG15^ and PDI-specific siRNA comparable to that of the control (Fig 5C, D and E). These data suggest that knockingdown endogenous PDI produces different effects on those two PrP mutants.

### PDI Involved in Mitochondrial Dysfunction Induced by Misfolded Prion

**Figure 6:**
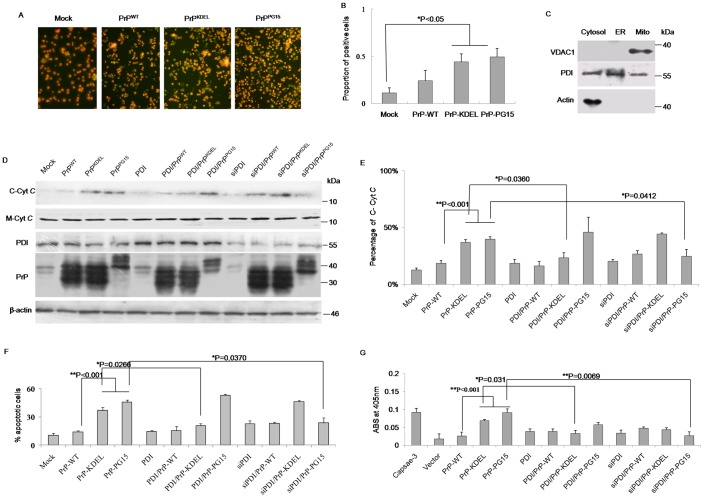
Assays of mitochondrial dysfunction in the cells expressing various PrP constructs under the condition of overexpression of PDI or knockdown of endogenous PDI. A. Images of the cells with abnormal mitochondrial transmembrane potentials under a fluorescence microscopy. Cells under apoptotic process are colored as green and alive cells are red (×200). B. Quantificational analysis of the percentages of apoptotic cells among the total cells. Data are calculated from three independent tests and presented as mean values ± SD. Statistical differences compared with controls are illustrated as *P<0.05. C. Qualitative evaluation of the prepared cell fractions with Western blots of VDAC1 (mitochondrial maker), PDI (ER maker) and actin (cytosol marker). D. Western blots for C-Cyt*C* outflux in the cytosol fractions and M-Cty*C* in the mitochondrial fraction of various preparations. The expressing levels of PDI and PrP in the cell lysates of various preparations were also evaluated with individual Western blots. E. Quantitative analysis of C-Cyt*C* with normalizing for mitochondrial membrane-associated Cyt *C*. Data are calculated from three independent tests and indicated as mean values ± SD. Statistical differences compared with controls are illustrated as *P<0.05 or **P<0.001 F. Cytoflowmetry analysis of the cells with mitochondrial dysfunction each preparation with a mitochondrial apoptosis detection kit. The percentages of apoptotic cells are shown in Y-axis. Data are calculated from three independent tests and indicated as mean values ± SD. Statistical differences compared with controls are illustrated as *P<0.05 or **P<0.001. G. Caspase-3 activity assays of various preparations with a commercial kit. 2 U/µl of recombinant human capsase-3 (marked as Caspase-3) supplied in the kit was used as the positive control. ABS indicates as absorbance at 405 nm. Data are calculated from three independent tests and indicated as mean values ± SD. Statistical differences compared with controls are illustrated as *P<0.05 or **P<0.001.

Organelle-specific initiation of cell death can be induced by various misfolded proteins based on different situations [20], [21]. To identify the involvement of mitochondrial dysfunction in apoptosis induced by those two PrP mutants, the mitochondrial transmembrane potentials (MTP) of the cells challenged with PrP mutants under conditions of overexpressing or downregulating PDI were evaluated. With the help of a commercial mitocapture kit that could distinguish the apoptotic cells (green) with mitochondria dysfunction from the live cells (red), more green-stained cells were observed in preparations of PrP^KDEL^ and PrP^PG15^, showing statistical significance after comparing percentages of apoptotic cells in preparations of PrP mutants with that of PrP^WT^ (Fig. 6A and B). Furthermore, levels of cytochrome *c* in cytoplasm (C-Cyt*C*) were measured with Western blots, which were represented as sequential events of MTP disruption leading to release of cytochrome *c* from mitochondria to cytoplasm. In parallel, the levels of membrane cytochrome *c* (M-Cyt*C*) were evaluated with Western blots. The cytosolic fraction from crude lysates was carefully prepared, which was marked with actin and PDI, but without voltage-dependent anion channel 1 (VDAC1, a biomarker for the outer membrane of mitochondria, Fig. 6C). Western blots showed comparable levels of M-Cyt*C* in all preparations (Fig 6D). Interestingly, in the group of transfection of PrPs alone, cells expressing PrP^KDEL^ or PrP^PG15^ showed stronger signals of C-Cyt*C* than that of PrP^WT^ and control, with statistical significance after the digitized gray values of C-Cyt*C* were normalized with that of individual M-Cyt*C* (Fig 6 D and E). Expression of PDI or PDI-specific siRNA alone seemed to not alter the level of C-Cyt*C* (Fig 6D). However, co-expression of PDI in the cells of PrP^KDEL^ partially reduced the level of C-Cyt*C* but not in that of PrP^PG15^, whereas co-expression of PDI-specific siRNA in cells of PrP^PG15^ remarkably decreased the level of C-Cyt*C* but not in PrP^KDEL^ (Fig 6D and E). Flow cytometry analysis revealed that the proportions of apoptotic cells in cells transfected with plasmids expressing PrP^KDEL^ and PrP^PG15^ alone, the cells co-expressing PrP^KDEL^ and PDI-specific siRNA and cells co-expressing PrP^PG15^ and PDI were significantly higher than in the other preparations (Fig 6F), which were consistent with the results of assays of cell viability and levels of C-Cyt*C*. Assays of the activities of caspase-3 identified higher enzyme activities in cells expressing either PrP^KDEL^ or PrP^PG15^, the cells co-challenged with PrP^KDEL^ and PDI-specific siRNA, as well as the cells co-expressing PrP^PG15^ and PDI (Fig 6F). Furthermore, the activities of caspase-3 in cells expressing natural fCJD associated PrP mutants (PrP-G114V and PrP-PG14) were significantly higher than that of mock, whereas the activity of caspase 3 in cells expressing PS1-KDEL remained unchanged (Supplemental Fig S3). Those data imply that, in addition to ER stress and ER-related apoptosis, mitochondrial dysfunction contributes to apoptosis caused by expressions of PrP mutants. Changes in cellular PDI levels result in distinct effects on mitochondria function in cells expressing different misfolded PrP mutants.

### *SNO-PDI in Prion Disease and Misfolded Prion Cells*

**Figure 7:**
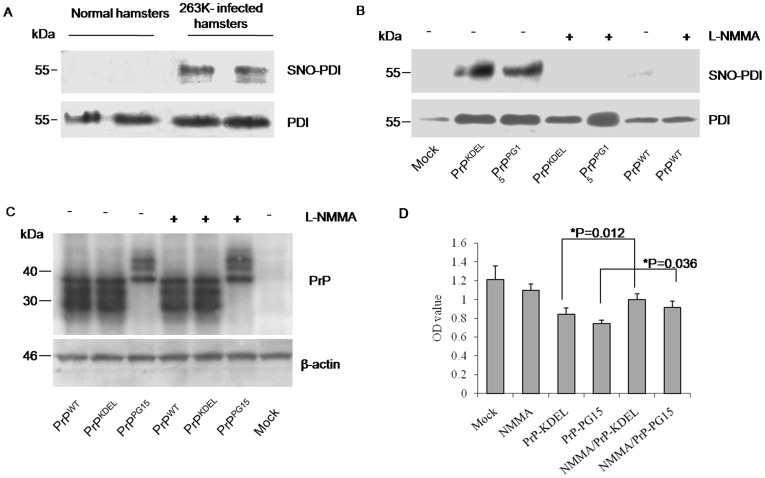
Detections of SNO-PDI in the brains of scrapie infected hamsters and in the cell models of misfolded prion proteins. A. Western blots for the PDI and SNO-PDI signals in hamsters’ brains. The biotinylated and immunoprecipitated by streptavidin-agarose beads proteins from brain homogenates of 263K infected or normal hamsters were separated in 15% SDS-PAGE and blotted with respective specific antibodies. B. Western blots for the PDI and SNO-PDI signals in the cell models in the presence (+) or absence (-) of L-NMMA. All cells were maintained for 48 h after transfection and then treated with 1 nM L-NMMA for 6 h. C. Evaluation of the influence of L-NMMA on the expressions of various PrP constructs by Western blots. Cell lysates were separated in 15% SDS-PAGE and blotted with respective antibodies. D. Cell viability. The results are calculated from three independent tests indicated as mean ± SD. Statistical differences compared with controls are illustrated as *P<0.05.

It has been suggested that PDIs can be modified by an S-nitrosylation process under continuous stress, which is involved in the pathogenesis of neurodegenerative disease [11]. To probe into the situation and the contribution of SNO-PDI in prion disease, levels of SNO-PDI in brain tissues of scrapie-263K-infected hamsters were screened with a biotin-switch assay. Accompanying the increases in PDI, a clear SNO-PDI signal was found in two infected brains but not in normal brains (Fig 7A), suggesting a disease-related phenomenon. To see this abnormal phenotype in cell models expressing PrP mutants, cell lysates transfected with different PrP constructs were subjected to SNO-PDI tests. Obvious SNO-PDI signals were detected in preparations of PrP^KDEL^ and PrP^PG15^, but not in that of control and PrP^WT^ (Fig 7B). In the presence of L-NMMA, an inhibitor for NO synthase (NOS), no SNO-PDI signal was detected in all preparations; meanwhile, neither the expression level of PDI (Fig 7B) nor that of PrP constructs showed remarkable change compared with those in the absence of L-NMMA (Fig 7C). It highlights that accumulations of PrP^KDEL^ and PrP^PG15^ in cytoplasm result in the formation of SNO-PDI possibly through activating cellular NOS. Further, the potential protective effect of reduction of SNO-PDI against the cytotoxicity of those two PrP mutants was evaluated. CCK-8 assays identified that decreased cell viabilities due to misfolded PrPs were partially, but significantly, recovered by the presence of L-NMMA (Fig. 7D). It seems that cytotoxicity of misfolded PrPs is partially due to inducement of SNO-PDI.

## Discussion

Although the real function of prion protein is still unknown, it is obvious that deposits of pathogenic or misfolded prion proteins could result in neuronal damage during the pathogenesis of prion diseases [22]. It has been well defined that various PrP mutants induce considerable neuron apoptosis *in vitro* and *in vivo*. Many molecules are identified as pro-apoptotic or anti-apoptotic agents in prion disease [23], [24]. In this artical, time-course studies revealed that PDI in the brains of infectious prion experimental animals is upregulated as the disease progression. We also for the first time provide PDI as a pleiotropic apoptosis regulator in cytotoxicity induced by two different misfolded prion proteins. In line with previous publications [25], overexpressions of PrP mutants in cultured cells can induce serious ER stress and activation of caspases, and eventually cause apoptosis. Caspase activation and upregulation of PDIs have been described previously in the brain of individuals affected by sporadic and variant CJD and murine scrapie [26], [27], which may represent an actively protective response of the host. By imitating the terminal pattern of PDIs in infected hamster brains, overexpression of PDI in cells expressing PrP mutants, especially PrP^KDEL^, partially eases the URP response and caspase-12 mediated ER apoptosis, which indicates that an increase in PDI to a certain degree releases stress accumulated in the ER. However, upregulation of endogenous protective factors, such as Grp58 and PDI, seems not enough to reverse the apoptotic fates of cells expressing PrP mutants and the damages in prion-infected brains. This is because severe pathological abnormalities and remarkable overexpression of PDI family members coexist in the brain tissues of scrapie-infected rodents at the terminal stages. In addition, persistent upregulation of PDI, Grp58 and UPR-associated Grp78/Bip has been detected in the pre-symptomatic phage of scrapie-infected hamsters, which may suggest that the protective effect of such factors is activated at an early stage [28].

Despite the protective effect of PDIs in prion disease, the question still remains whether they are involved in apoptotic pathology at the terminal stage. A pathway of PDIs-associated apoptosis for misfolded proteinS has been identified in Huntington’s disease. Excess of PDI can recruit the pro-apoptotic factor in mitochondria-associated-membrane (MAM) and can result in a cascade of caspases [10]. In previous work, we proposed that ER stress in cells expressing various PrP mutants within the transmembrane region [12] and with insertional octarepeats [15], accompanies increases in caspase-3 activity. Moreover, in this study we repeatedly found evidences of an increase in the Bax/Bcl-2 ratio and the presences of mitochondrial dysfunction in cells expressing misfolded prion proteins, accompanied by alteration of PDI. Activation of caspase-3 also has been detected in the brains of scrapie-infected hamsters. These observations may highlight an intrinsic link between upregulation of PDI and mitochondrial dysfunction in the pathology of prions. On the other hand, some studies have also proposed that ER stress or ubiquitin-proteasome system malfunction may not be the direct causative mechanism for some genetic human prion disease patients and transgenic mice [29], [30]. In XBP-1 KO mice, impaired ER stress is not able to alert prion replication and its relative pathogenesis [31]. Quaglio, et al failed to detect ER stress or proteasome dysfunction in transgenic mice with PrP mutants [29]. One possibility for this atypical result is that those special sub-populations of PrP mutants are invisible to the checkpoint of ER-based quality control machinery, and they selectively experience post-ER metabolism, termed Golgi-based quality control (Golgi-QC) [16]. Besides ER retention, PDI-associated mitochondrial malfunction and the disturbing localization of mutated PrP in the Golgi body, especially PrP-KDEL, have also been observed in this study. Therefore, in the process of mutated PrP multimerization, abnormal regulation of ER-retained chaperones, such as PDIs, may not only be involved in ER stress, but also in other pathways, including Golgi-QC.

*S*-nitrosylated modification of PDI is identified in some neurodegenerative diseases and is proposed to link with misfolded proteins in Parkinson’s disease and Alzheimer’s disease [11]. Our data here illustrate obvious SNO-PDI signals in brain tissues of scrapie-infected animals, which are undetectable in normal controls. Our data indicate *S*-nitrosylation of PDI may be a common phenotype in neurodegenerative diseases. In parallel, marked formations of SNO-PDI are observed in cell models of mutated prion proteins. Blocking the activity of cellular NOS can not only significantly inhibit formation of SNO-PDI but also rescue the cytotoxicity induced by misfolded prion proteins. Those data highlight the formation of SNO-PDI induced by TSE agents or highlight how misfolded PrP proteins plays critical role in their cytotoxicity. Based on published documents, it is reasonable to presume that the expressions of mutated PrP proteins can cause a dramatic flux of ER calcium content to cytoplasm [3], which may generate reactive oxygen species (ROS ) and activate NOS in cells [18]. The release of excessive NO triggers the formation of SNO-PDI [11], [32].

PDI seems to be involved in regulation of misfolded prion protein in a complicated and pleiotropic manner. At an early stage, PDI provides with protective activity to eliminate misfolded proteins; at the late stage, perturbation of oxidative homeostasis and its inducement of SNO-PDI facilitate PDI-associated apoptosis during prion infection. The pleiotropic roles of PDI are also illustrated in the regulation of cytotoxicity of two distinct mutated PrP constructs in cell models. PrP^KDEL^ caused cytotoxicity, a normal PrP molecular organization with ER-adhering orientation mimicking PrP mutants within the transmembrane region [12], [33], mostly triggering the abnormal accumulation of PrP in ER and the malfunction of ER stress. Coexpression of PDI can partially reverse an increase in the Bax/Bcl-2 ratio and cytotoxicity induced by PrP^KDEL^, whereas downregulation of cellular PDI aggravates those effects. However, PrP^PG15^, an abnormal PrP molecular organization with octarepeat insertion representing a series of genetic CJD-associated insertional mutants [15], [33], is seemingly involved in both ER stress and mitochondrial apoptosis. The cytotoxitic effect of PrP^PG15^ did not seem to be improved by co-expression of PDI, even when mitochondrial dysfunction was worsened, whereas down-regulation of cellular PDI clearly amended PrP^PG15^-induced mitochondrial dysfunction and cytotoxicity. It might reflect a multi-faceted event of cellular regulation of PDI, possibly related to the refined structural diversities of prion proteins, which also suggests PDI as a target for easing the neuronal damage and toxic pathology of prion disease. Nevertheless, both PrP constructs have the ability to induce the formation of SNO-PDI eventually, which probably contributes to cytotoxicity of misfolded PrP proteins in cultured cells and to irreversible neuron damage in prion infections at a late stage.

## Supporting Information

Figure S1 Co-localizations of the expressed PrPs and Golgi compartment in 293-T cells. Cells receiving various PrP constructs were immunocytochemically stained 48 h after transfection. The images of PrP (green), Golgi (red), DAPI (blue) and merge are monitored under a confocal microscopy and indicated above. (×1000).
(TIF)

Figure S2 Influences of expressions of PS1-KDEL, PrP-G114V and PrP-PG14 on the cell viability and the relevant cellular factors. A. Cell viabilities assayed by CCK-8 kit. Each group is assigned to three repeating parallel units and measured with a spectrophotometer under 450 nm. The results are indicated as mean ± SD. Statistical differences compared with controls are illustrated as *P<0.05. B. Western blots. Cell lysates were separated in 15% SDS-PAGE and the specific immunoblots for Grp78, pro-caspase12, PDI, pro-caspase3 and PrP were detected with individual antibodies. C. Quantitative analysis of each gray numerical value of Grp78, pro-caspase12, PDI, pro-caspase3 vs that of individual β-actin. The results are calculated from three independent tests presented as mean ± SD. Statistical differences are illustrated as *P<0.05.
(TIF)

Figure S3 Caspase-3 activity assays of various preparations with a commercial kit. 2 U/µl of recombinant human capsase-3 (marked as Caspase-3) supplied in the kit was used as the positive control. ABS indicates as absorbance at 405 nm. Test was repeated for three times. Data are shown as mean values ± SD. Statistical differences are illustrated as *P<0.05.
(TIF)

## References

[1] Prusiner SB (1998). The prion diseases.. Brain Pathol.

[2] Hetz C, Glimcher LH (2009). Fine-tuning of the unfolded protein response: Assembling the IRE1alpha interactome.. Mol Cell.

[3] Torres M, Castillo K, Armisen R, Stutzin A, Soto C (2010). Prion protein misfolding affects calcium homeostasis and sensitizes cells to endoplasmic reticulum stress.. PLoS One.

[4] Kim BH, Lee HG, Choi JK, Kim JI, Choi EK (2004). The cellular prion protein (PrPC) prevents apoptotic neuronal cell death and mitochondrial dysfunction induced by serum deprivation.. Brain Res Mol Brain Res.

[5] Rutkowski DT, Wu J, Back SH, Callaghan MU, Ferris SP (2008). UPR pathways combine to prevent hepatic steatosis caused by ER stress-mediated suppression of transcriptional master regulators.. Dev Cell.

[6] Hampton RY (2000). ER stress response: getting the UPR hand on misfolded proteins.. Curr Biol.

[7] Turano C, Coppari S, Altieri F, Ferraro A (2002). Proteins of the PDI family: unpredicted non-ER locations and functions.. J Cell Physiol.

[8] Tanaka S, Uehara T, Nomura Y (2000). Up-regulation of protein-disulfide isomerase in response to hypoxia/brain ischemia and its protective effect against apoptotic cell death.. J Biol Chem.

[9] Hetz CA (2007). ER stress signaling and the BCL-2 family of proteins: from adaptation to irreversible cellular damage.. Antioxid Redox Signal.

[10] Hoffstrom BG, Kaplan A, Letso R, Schmid RS, Turmel GJ (2010). Inhibitors of protein disulfide isomerase suppress apoptosis induced by misfolded proteins.. Nat Chem Biol.

[11] Uehara T, Nakamura T, Yao D, Shi ZQ, Gu Z (2006). S-nitrosylated protein-disulphide isomerase links protein misfolding to neurodegeneration.. Nature.

[12] Wang X, Shi Q, Xu K, Gao C, Chen C (2011). Familial CJD associated PrP mutants within transmembrane region induced Ctm-PrP retention in ER and triggered apoptosis by ER stress in SH-SY5Y cells.. PLoS One.

[13] Guo YJ, Wang XF, Han J, Zhang BY, Zhao WQ (2008). A patient with Creutzfeldt-Jakob disease with an insertion of 7 octa-repeats in the PRNP gene: molecular characteristics and clinical features.. Am J Med Sci.

[14] Han L, Wan YZ, Han J, Chen L, Sun L (2007). Preliminary analyses for influence of mutant PrPs with different number of octapeptide.. Zhonghua Shi Yan He Lin Chuang Bing Du Xue Za Zhi.

[15] Xu K, Wang X, Shi Q, Chen C, Tian C (2011). Human prion protein mutants with deleted and inserted octarepeats undergo different pathways to trigger cell apoptosis.. J Mol Neurosci.

[16] Ashok A, Hegde RS (2009). Selective processing and metabolism of disease-causing mutant prion proteins.. PLoS Pathog.

[17] Castilla J, Gutierrez-Adan A, Brun A, Pintado B, Salguero FJ (2005). Transgenic mice expressing bovine PrP with a four extra repeat octapeptide insert mutation show a spontaneous, non-transmissible, neurodegenerative disease and an expedited course of BSE infection.. FEBS Lett.

[18] An R, Dong C, Lei Y, Han L, Li P (2008). PrP mutants with different numbers of octarepeat sequences are more susceptible to the oxidative stress.. Sci China C Life Sci.

[19] Martin SF, Herva ME, Espinosa JC, Parra B, Castilla J (2006). Cell expression of a four extra octarepeat mutated PrPC modifies cell structure and cell cycle regulation.. FEBS Lett.

[20] Boyce M, Yuan J (2006). Cellular response to endoplasmic reticulum stress: a matter of life or death.. Cell Death Differ.

[21] Ferri KF, Kroemer G (2001). Organelle-specific initiation of cell death pathways.. Nat Cell Biol.

[22] Chiesa R, Piccardo P, Biasini E, Ghetti B, Harris DA (2008). Aggregated, wild-type prion protein causes neurological dysfunction and synaptic abnormalities.. J Neurosci.

[23] Liberski PP, Brown DR, Sikorska B, Caughey B, Brown P (2008). Cell death and autophagy in prion diseases (transmissible spongiform encephalopathies).. Folia Neuropathol.

[24] Kristiansen M, Messenger MJ, Klohn PC, Brandner S, Wadsworth JD (2005). Disease-related prion protein forms aggresomes in neuronal cells leading to caspase activation and apoptosis.. J Biol Chem.

[25] Nunziante M, Ackermann K, Dietrich K, Wolf H, Gadtke L (2011). Proteasomal dysfunction and ER stress enhance trafficking of prion protein aggregates through the secretory pathway and increase accumulation of PrPSc..

[26] Yoo BC, Krapfenbauer K, Cairns N, Belay G, Bajo M (2002). Overexpressed protein disulfide isomerase in brains of patients with sporadic Creutzfeldt-Jakob disease.. Neurosci Lett.

[27] Hetz C, Russelakis-Carneiro M, Maundrell K, Castilla J, Soto C (2003). Caspase-12 and endoplasmic reticulum stress mediate neurotoxicity of pathological prion protein.. EMBO J.

[28] Hetz C, Russelakis-Carneiro M, Walchli S, Carboni S, Vial-Knecht E (2005). The disulfide isomerase Grp58 is a protective factor against prion neurotoxicity.. J Neurosci.

[29] Quaglio E, Restelli E, Garofoli A, Dossena S, De Luigi A (2011). Expression of mutant or cytosolic PrP in transgenic mice and cells is not associated with endoplasmic reticulum stress or proteasome dysfunction.. PLoS One.

[30] Unterberger U, Hoftberger R, Gelpi E, Flicker H, Budka H (2006). Endoplasmic reticulum stress features are prominent in Alzheimer disease but not in prion diseases in vivo.. J Neuropathol Exp Neurol.

[31] Hetz C, Lee AH, Gonzalez-Romero D, Thielen P, Castilla J (2008). Unfolded protein response transcription factor XBP-1 does not influence prion replication or pathogenesis.. Proc Natl Acad Sci U S A.

[32] Garthwaite J, Charles SL, Chess-Williams R (1988). Endothelium-derived relaxing factor release on activation of NMDA receptors suggests role as intercellular messenger in the brain.. Nature.

[33] Shi Q, Dong XP (2011). (Ctm) PrP and ER stress: A neurotoxic mechanism of some special PrP mutants.. Prion.

