# Supplementary figures and images for: Protein Disulfide Isomerase Regulates Endoplasmic Reticulum Stress and the Apoptotic Process during Prion Infection and PrP Mutant-Induced Cytotoxicity
Source: PLoS One. 2012 Jun 7;7(6):e38221. doi: 10.1371/journal.pone.0038221 (PMC3369880; doi:10.1371/journal.pone.0038221)

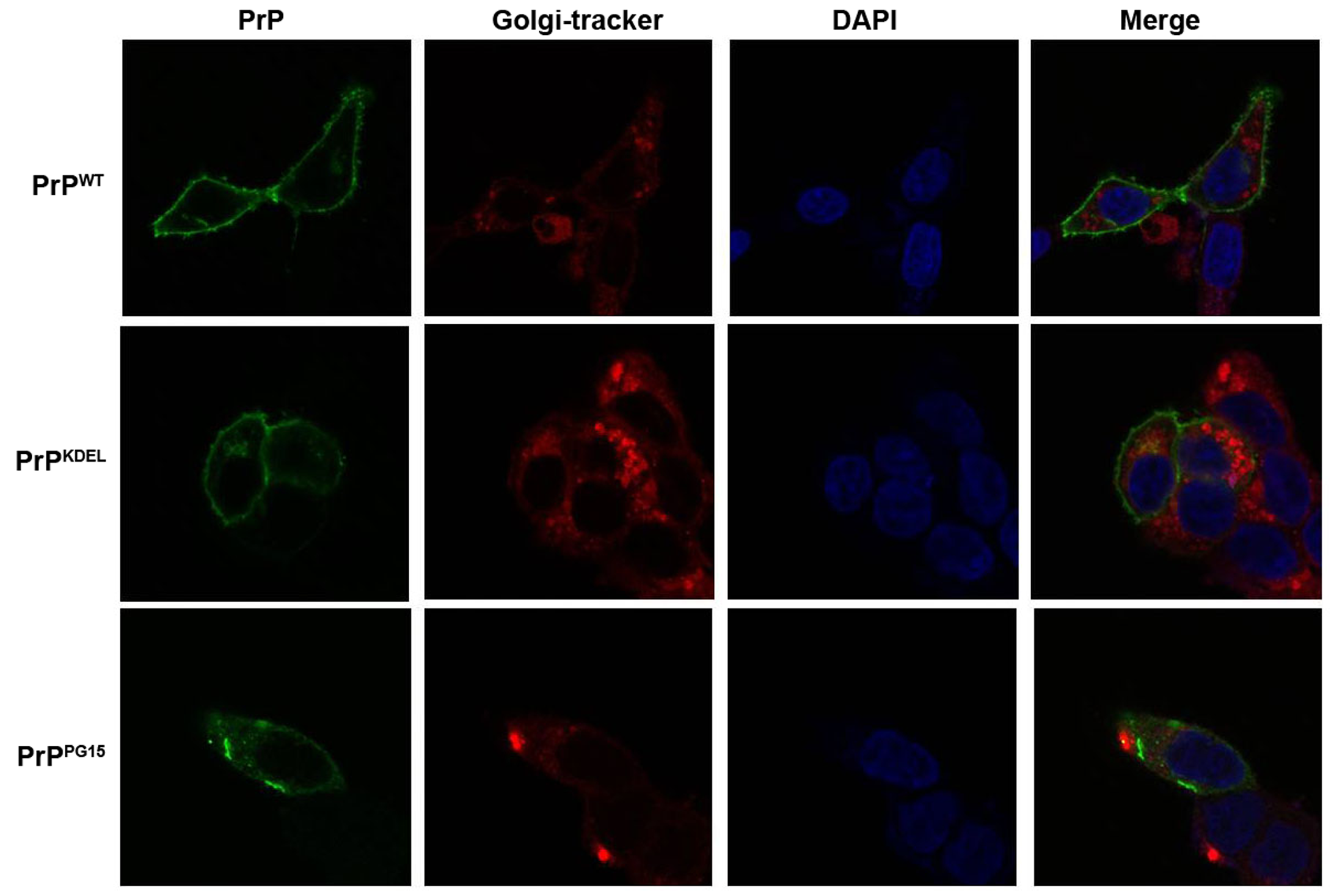

Supplement: Figure S1 — Co-localizations of the expressed PrPs and Golgi compartment in 293-T cells. Cells receiving various PrP constructs were immunocytochemically stained 48 h after transfection. The images of PrP (green), Golgi (red), DAPI (blue) and merge are monitored under a confocal microscopy and indicated above. (×1000). (TIF) [file pone.0038221.s001.tif]

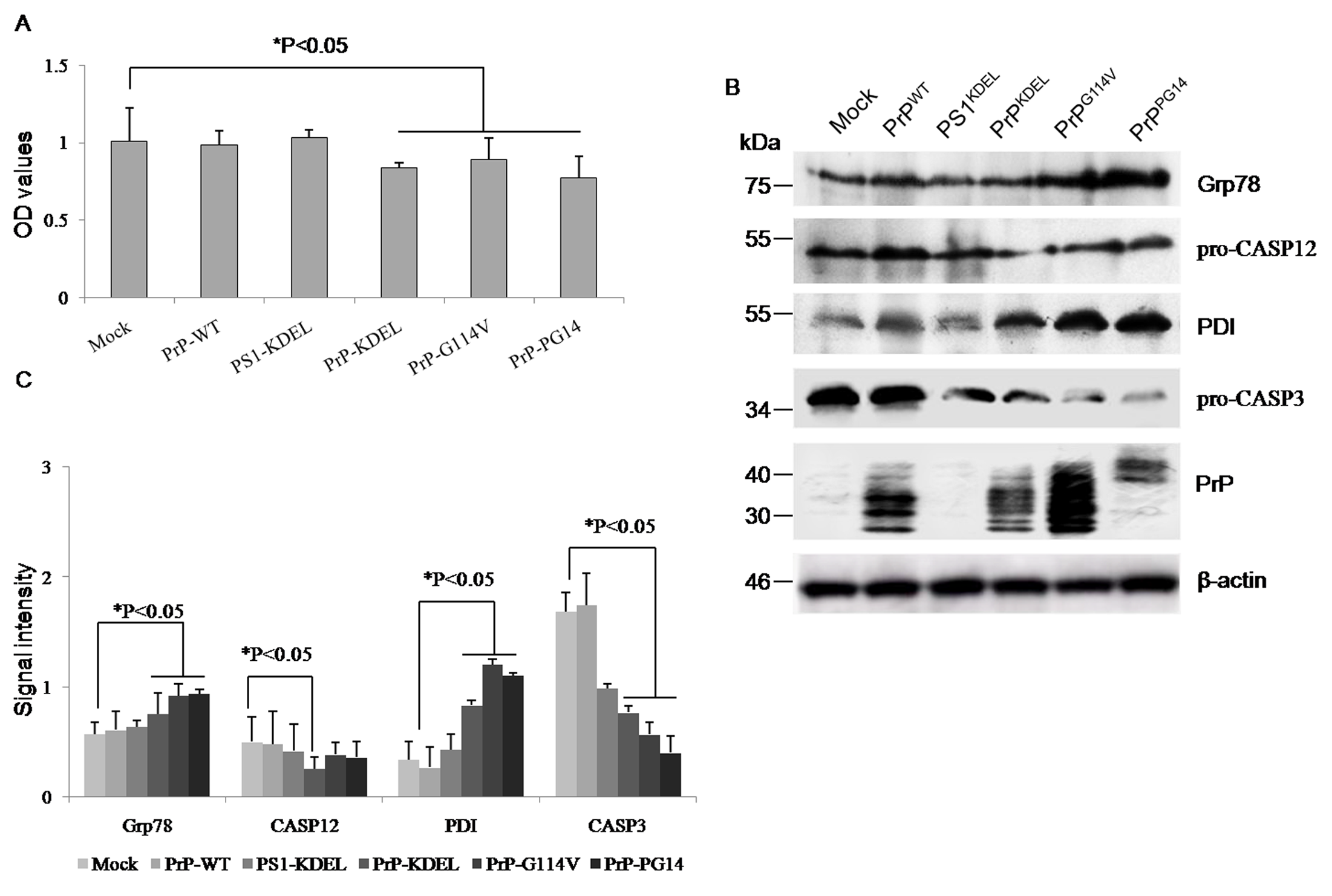

Supplement: Figure S2 — Influences of expressions of PS1-KDEL, PrP-G114V and PrP-PG14 on the cell viability and the relevant cellular factors. A. Cell viabilities assayed by CCK-8 kit. Each group is assigned to three repeating parallel units and measured with a spectrophotometer under 450 nm. The results are indicated as mean ± SD. Statistical differences compared with controls are illustrated as *P<0.05. B. Western blots. Cell lysates were separated in 15% SDS-PAGE and the specific immunoblots for Grp78, pro-caspase12, PDI, pro-caspase3 and PrP were detected with individual antibodies. C. Quantitative analysis of each gray numerical value of Grp78, pro-caspase12, PDI, pro-caspase3 vs that of individual β-actin. The results are calculated from three independent tests presented as mean ± SD. Statistical differences are illustrated as *P<0.05. (TIF) [file pone.0038221.s002.tif]

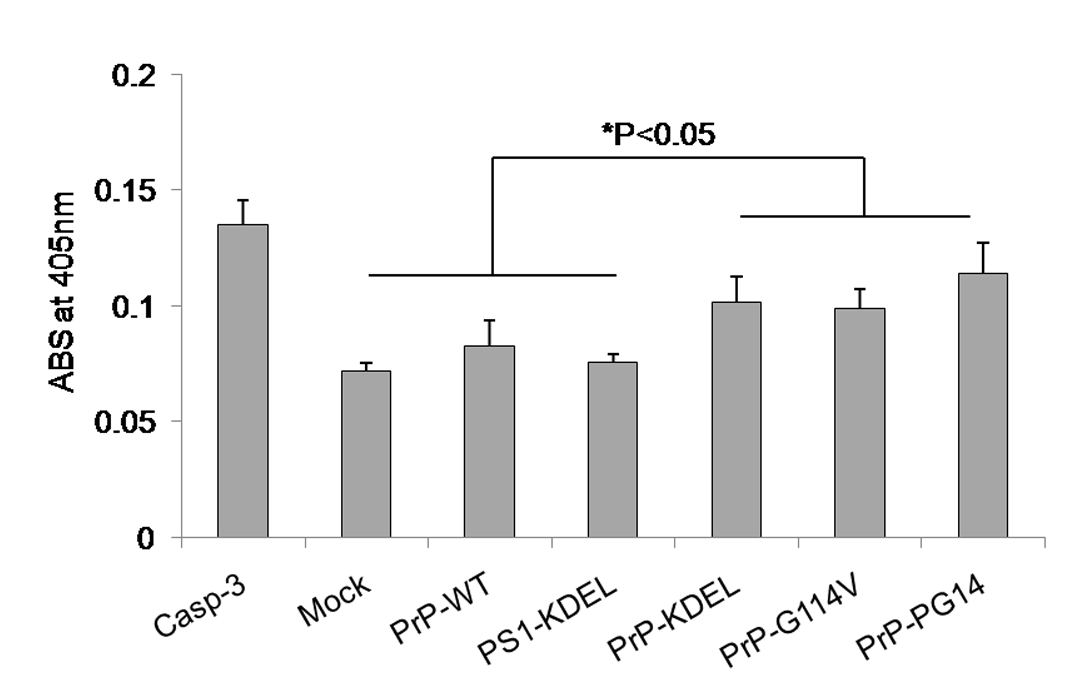

Supplement: Figure S3 — Caspase-3 activity assays of various preparations with a commercial kit. 2 U/µl of recombinant human capsase-3 (marked as Caspase-3) supplied in the kit was used as the positive control. ABS indicates as absorbance at 405 nm. Test was repeated for three times. Data are shown as mean values ± SD. Statistical differences are illustrated as *P<0.05. (TIF) [file pone.0038221.s003.tif]
